# Anti-human platelet antigen (HPA)-1a antibodies may affect trophoblast functions crucial for placental development: a laboratory study using an in vitro model

**DOI:** 10.1186/s12958-017-0245-6

**Published:** 2017-04-21

**Authors:** Mariana Eksteen, Gøril Heide, Heidi Tiller, Yan Zhou, Nora Hersoug Nedberg, Inigo Martinez-Zubiaurre, Anne Husebekk, Bjørn R. Skogen, Tor B. Stuge, Mette Kjær

**Affiliations:** 1 Immunology research group, Department of Medical Biology, Faculty of Health Sciences, UiT - The Arctic University of Norway, Tromsø, Norway; 2 Department of Obstetrics and Gynecology, University Hospital of North Norway, Tromsø, Norway; 3 Department of Obstetrics, Gynecology & Reproductive Sciences, University of California, San-Francisco, CA USA; 4 Prophylix Pharma AS, Tromsø, Norway; 5 Bone and Joint research group, Department of Clinical Medicine, UiT- The Arctic University of Norway, Tromsø, Norway; 6 Department of Laboratory Medicine, University Hospital of North Norway, Tromsø, Norway

**Keywords:** Alloimmunization, HPA-1a, Anti-HPA-1a antibodies, Trophoblast cells, Placental development, αVβ3, Vitronectin receptor, Fetal and neonatal alloimmune thrombocytopenia

## Abstract

**Background:**

Fetal and neonatal alloimmune thrombocytopenia (FNAIT) is a bleeding disorder caused by maternal antibodies against paternal human platelet antigens (HPAs) on fetal platelets. Antibodies against HPA-1a are accountable for the majority of FNAIT cases. We have previously shown that high levels of maternal anti-HPA-1a antibodies are associated with clinically significant reduced birth weight in newborn boys. Chronic inflammatory placental lesions are associated with increased risk of reduced birth weight and have previously been reported in connection with FNAIT pregnancies. The HPA-1a epitope is located on integrin β3 that is associated with integrin αIIb (the fibrinogen receptor) on platelets and megakaryocytes. Integrin β3 is also associated with integrin αV forming the αVβ3 integrin heterodimer, the vitronectin receptor, which is expressed on various cell types, including trophoblast cells. It is therefore thinkable that maternal anti-HPA-1a antibodies present during early pregnancy may affect placenta function through binding to the HPA-1a antigen epitope on invasive throphoblasts. The aim of the study was to examine whether interaction of a human anti-HPA-1a monoclonal antibody (mAb) with HPA-1a on trophoblast cells affect adhesion, migration and invasion of extravillous trophoblast cells.

**Methods:**

An in vitro model with human anti-HPA-1a mAb, clone 26.4, and the first trimester extravillous trophoblast cell line HTR8/SVneo was employed. The xCELLigence system was utilized to assess the possible effect of anti-HPA-1a mAb on adhesion and migration of HTR8/SVneo cells. Specially designed chambers precoated with Matrigel were used to assess the effect on the invasive capacity of cells.

**Results:**

We found that human anti-HPA-1a mAb 26.4 partially inhibits adhesion and migratory capacity of HTR8/SVneo cells.

**Conclusions:**

Our findings suggest that anti-HPA-1a antibodies may affect trophoblast functions crucial for normal placental development. Future studies including primary throphoblast cells and polyclonal anti-HPA-1a antibodies are needed to confirm these results.

## Background

Fetal and neonatal alloimmune thrombocytopenia (FNAIT) is caused by maternal antibodies against alloantigens on fetal platelets. It is a rare, but potentially life threatening disorder with intracranial hemorrhage (ICH) as the most severe complication. Severe gastrointestinal and pulmonary hemorrhages have also been reported [1]. Antibodies against human platelet antigen (HPA)-1a are accountable for nearly 85% of FNAIT cases [2]. The frequency of FNAIT due to anti-HPA-1a antibodies is around one per 1100 live births [2, 3]. We have previously found that high levels of maternal anti-HPA-1a antibodies are associated with clinically significant reduced birth weight in newborn boys [4]. A similar observation was made in an international multicenter study of FNAIT-associated ICH, showing that 23% of neonates with ICH were small for gestational age [5]. Chronic inflammatory placental lesions like chronic villitis and intervillositis have been reported in association with FNAIT cases [6] and such placental lesions are known to be associated with increased risk of fetal growth restriction.

Integrin β3, carrying the HPA-1 antigen epitope, is expressed on platelets and megakaryocytes as part of αIIbβ3 integrin heterodimer, the fibrinogen receptor. Integrin β3 is also associated with αV integrin forming integrin heterodimer αVβ3, also known as vitronectin receptor. The vitronectin receptor is expressed on various cell types, including trophoblast cells [7–9].

During early pregnancy, a population of trophoblast cells differentiates into highly invasive extravillous trophoblasts (EVT). EVT invade the decidualized endometrium reaching the inner third of the myometrium, and migrate along the spiral arteries remodeling them into large diameter low resistance vessels [10]. EVT migration and invasion into the uterus continues until mid-gestation and is regulated by various factors of both maternal and embryonic origin [11]. Impaired trophoblast invasion and insufficient remodeling of placental spiral arteries are common histopathological findings in placentas from pregnancies complicated by preeclampsia and low birth weight [12, 13].

During migration and invasion, EVT cells undergo integrin switch and upregulate expression of adhesion molecules on cell surface, including the αVβ3 [8, 14]. The important role of αVβ3 in mediating migration and invasion of primary cytotrophoblasts (CTB) was demonstrated in vitro [8, 15]. It has therefore been speculated that anti-HPA-1a antibodies may affect placental development [4]. Anti-HPA-1a antibodies can bind HPA-1a on αVβ3 expressed on trophoblast cells [9, 16], and we hypothesize that this binding may affect EVT invasion, spiral artery remodeling, and in turn lead to reduced placental function.

The objective of this study was to test whether anti-HPA-1a antibodies affect adhesion, migration and invasive capacity of EVT cells. For functional experiments we used an experimental in vitro model with human recombinant anti-HPA-1a monoclonal antibody (mAb), clone 26.4 [16], and a first trimester human EVT-derived cell line, HTR8/SVneo [17].

## Methods

### Cell culture

Human first trimester extravillous trophoblast-derived cell line, HTR8/SVneo, was kindly provided by Charles Graham (Department of Anatomy and Cell Biology at Queen’s University, Kingston, ON, Canada). The cell line was generated by immortalization of primary villous explant culture from first trimester human placenta (8–10 WG) with SV40 virus [17]. HTR8/SVneo is a hypotriploid cell line (3n-) [18]. Cells were cultured in RPMI-1640 (Sigma-Aldrich, St. Louis, MO), supplemented with 10% FBS (Lonza, Basel, Switzerland), 100 U/ml penicillin, 100 U/ml streptomycin (Lonza) and maintained at 37 °C, in a 5% CO_2_ humidified atmosphere_._ The cells were grown to 70–80% confluency and passaged 24 h prior to experiments. The cells were detached by incubation with 2 mM EDTA in PBS for 5 min at 37 °C.

### Antibodies

A recently developed human recombinant anti-HPA-1a IgG1 mAb (clone 26.4) [16] was used to explore the effect on invasive trophoblast cells. Murine anti-human αVβ3 mAb, clone LM609 (Millipore, Billerica, MA) was used as positive control for cell functional studies. Sodium azide from LM609 sample was removed by buffer exchange with PBS using PD SpinTrap G-25 (GE Healthcare, Little Chalfont, UK). Integrin β3 was detected using murine mAb, clone SZ21, HPA-1-reactive [19] (Dako, Glostrup, Denmark) and rabbit mAb, clone EPR2417Y (Abcam, Cambridge, UK). Alexa Fluor 488-conjugated goat anti-mouse and goat anti-human antibodies (Invitrogen, Carlsbad, CA) were used as secondary antibodies in flow cytometry experiments. Human myeloma plasma IgG1 (Sigma) and murine IgG1 (Beckman Coulter, Brea, CA) were used as isotype controls. Horseradish peroxidase (HRP)-conjugated goat anti-rabbit IgG (Thermo Scientific, Waltham, MA) was used as a detection antibody in the western blot experiment.

### Western blot

Platelets from an HPA-1aa-genotyped donor (16 × 10^8^ cells) and HTR8/SVneo cells (20 × 10^6^ cells) were lysed using 3 ml RIPA buffer (Sigma) in the presence of protease inhibitor (cOmplete Tablets Mini EDTA-free, Roche Diagnostics, Basel, Switzerland). Twelve microliters of platelet lysate diluted 1:1000 and 12 μl of HTR8/SVneo cell lysate were reduced and separated in a 4–12% SDS polyacrylamide gel (Life Technologies, Carlsbad, CA). Electrophoresed samples were transferred to a PVDF membrane (Life Technologies). Nonspecific binding sites were blocked by Super blocking buffer (Thermo Scientific) containing 0.05% Tween 20 and 0.2% goat IgG (Thermo Scientific) for 1 h. Primary and secondary antibodies were diluted in Super blocking buffer containing 0,05% Tween 20. The PVDF membrane was incubated overnight at 4 °C with rabbit anti-β3 antibody diluted 1:2000 (clone EPR2417Y). After a washing step, the membrane was incubated with HRP-conjugated goat anti-rabbit IgG diluted 1:1000 for 1 h at RT followed by a washing step with PBS 0.05% Tween 20. The membrane was covered by 3 ml of Supersignal West Femto Maximum Sensitivity Substrate (Thermo Scientific) and left for 5 min in the dark at RT. Integrin β3 was visualized using the luminescent image analyzer ImageQuant LAS 4000 (GE Healthcare, Little Chalfont, UK). Integrin αVβ3 purified from human placenta was used as a positive control (Millipore, Billerica, MA). The expected β3 subunit band is of approximately 90–110 kDa.

### Flow cytometry

To stain cell surface membrane integrins, the HTR8/SVneo cells were harvested, washed and re-suspended in PBS 0.2% bovine serum albumin, and incubated 10 min at RT with unconjugated mouse anti-human β3 (clone SZ21) or human anti-HPA-1a (clone 26.4) mAbs. Mouse and human IgG1 were used as isotype controls. After a washing step, cells were stained with Alexa Fluor 488-conjugated goat anti-mouse and goat anti-human antibodies respectively, and analyzed by flow cytometry (Canto, Becton Dickinson, Franklin Lakes, NJ). The acquired data was analyzed using FlowJo software (TreeStar, Ashland, OR, USA).

### HPA-1 genotyping

The DNA and RNA from HTR8/SVneo cells and donor samples were isolated and used for HPA-1 genotyping by TaqMan 5′ nuclease assay as described previously [20, 21].

### Y-chromosome DNA test

The DNA isolated from HTR8/SVneo cells was used for Y-chromosome DNA test by TaqMan 5′ nuclease assay. The primers used for the assay were described previously [22] and FAM-labelled probe was designed in house.

### Monitoring cell adhesion and migration

Cell adhesion and migration were monitored in real time using the xCELLigence system (Roche Applied Science, Penzberg, Germany) [23]. For determining the rate of cell adherence, E-plate 16 assemblies were coated with human vitronectin (Promega, Madison, WI) by incubating 1 μg/ml solution in 100 μl volume for 1 h at 37 °C. The wells were washed twice with PBS before 50 μl complete medium was added and the background measurements recorded. The cells were seeded at 20,000 cells/well in a 40 μl volume. From a solution of 200 μg antibodies/ml PBS, 10 μl were added to each well (human IgG1 as negative control, 26.4 and LM609). Each plate was then assembled on the RTCA DP analyzer, and data were gathered at 5-min intervals for 7 h at 37 °C, in a 5% CO_2_ humidified atmosphere.

Cell migration was monitored using specially designed CIM-plate 16 with 8-μm pores. The sensor side (bottom side) of each well of the upper chamber was coated with human vitronectin by incubating 30 μl of the 1 μg/ml solution for 30 min at RT. The lower chambers were filled with medium containing 10% FBS, used as chemoattractant. The upper chambers were filled with serum-free medium (50 μl/well), and the plate was incubated at 37 °C in 5% CO_2_ for 1 h. After recording background measurements, the cells were seeded into the upper chamber at 40,000 cells in 40 μl per well and 10 μl of 200 μg/ml antibodies in PBS were added. The plate was then incubated for 30 min at RT, assembled on the RTCA DP analyzer and data collected every 15 min for 24 h at 37 °C, in a 5% CO_2_ humidified atmosphere. The obtained data were analyzed using the RTCA 1.2 software supplied with the instrument.

### Invasion assay

Cell invasion was evaluated using BD BioCoat Matrigel Invasion Chambers (BD Biosciences). The Chambers (24 well Plate 8 Micron with Control inserts) were prepared following the manufacturer’s instructions. The RPMI 1640 medium with 5% FBS was used as chemoattractant. HTR8/SVneo cells were seeded into each insert at 40,000 cells/well in a 180 μl volume in serum free medium and 20 μl of 200 μg/ml antibodies in PBS were added (total antibody concentration of 20 μg/ml). The plate was incubated for 48 h at 37 °C, in a 5% CO_2_ humidified atmosphere. After incubation, the non-invading cells were scrubbed from the upper part of the inserts by a cotton swab.

The invaded cells were measured by the MTT (3-[4,5-dimethylthiazol-2-yl]-2,5 diphenyl tetrazolium bromide) assay. The MTT (Sigma) at 5 mg/ml in RPMI 1640 medium without phenol red, was diluted 1:10 and 350 μl of the dilution was added to each clean well. The inserts were transferred to MTT solution and incubated for 2 h at 37 °C, in a 5% CO_2_ humidified atmosphere. Next, the inserts were transferred into clean wells with 220 μl of 0.04 M HCl in pure isopropanol and incubated for 5 min at RT. The inserts were removed and the solution transferred to centrifuge tubes and centrifuged for 2 min at 16,000 x g. Of the solution, 100 μl was transferred into a 96-well microtiter plate and absorption at 560 nm was measured by an ELISA- reader (Multiskan Ex, Thermo Scientific).

### Statistical Analysis

A one-way analysis of variance (ANOVA) in SPSS software (SPSS Inc., Chicago, IL, USA) was used to analyze adhesion, migration and invasion experimental data. A *P*-value of < 0.05 was considered significant. Sigma Plot 13 software (San Jose, CA) was used to present the data.

## Results

### A human anti-HPA-1a mAb 26.4 binds HPA-1a epitope on HTR8/SVneo cells

**Fig. 1:**
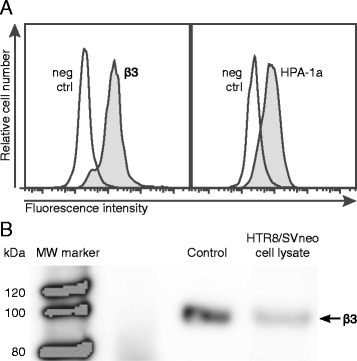
Expression of integrin β3 and HPA-1a on HTR8/SVneo cells. **a** Flow cytometric analysis of cells incubated with murine anti-human β3 HPA-1-reactive mAb SZ21 and human HPA-1a-specific mAb 26.4, or isotype control. **b** Detection of integrin β3 in lysed HTR8/SVneo cells by Western blot. Integrin αVβ3 isolated from human placenta used as control. Integrin β3 detected as a band of about 100 kDa in the control and cell lysate. Western blot image was spliced to rearrange the order of samples within one experiment. Dashed lines indicate where the images were joined. Figure A and B are representative for at least three independent experiments

Integrin β3 expression by HTR8/SVneo cells was assessed with Western blot and flow cytometry techniques. Both techniques demonstrated expression of integrin β3 by HTR8/SVneo cells (Fig. 1a and b). The cells expressed αV, but were negative for αIIb (data not shown), indicating that HTR8/SVneo cells express β3 integrin only in association with αV integrin. Next, HTR8/SVneo cells were genotyped HPA-1ab. Finally, flow cytometry analysis demonstrated that human anti-HPA-1a mAb bound to intact HTR8/SVneo cells (Fig. 1a).

### A human anti-HPA-1a mAb 26.4 partially inhibits adhesion and migratory capacity of HTR8/SVneo cells

**Fig. 2:**
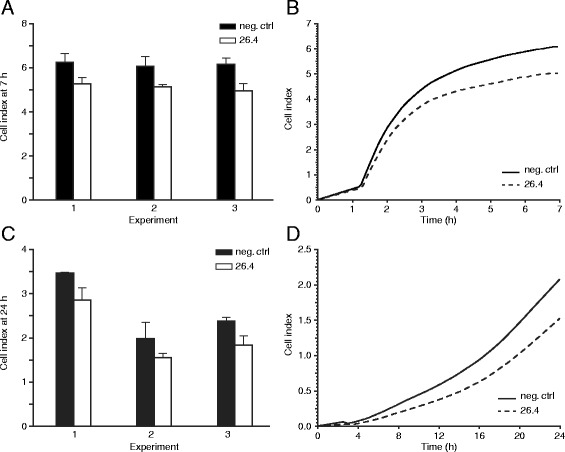
Effect of anti-HPA-1a antibodies on EVT adhesion and migration. HTR8/SVneo cells were plated on E-plate 16 (for adhesion experiments) and CIM-plate 16 (for migration experiments) in the presence of mAb 26.4 or human IgG1 as a negative control. Cell adhesion was monitored using xCELLigence system over 7 h, and cell migration over 24 h. The samples were run in triplicates and experiments repeated three times each. Each column represent the range in cell index of one sample run in triplicate. Results presented in the graphs (distinguished by experiment due to high variations in cell index between the experiments) are from three independent experiments: **a** adhesion; **c** migration. Plots visualize cell spreading and attachment in real time: **b** adhesion; **d** migration. Plots are representative for three independent experiments

The effect of a mAb 26.4 on trophoblast cell adhesion and migration was explored using the xCELLigence system. mAb 26.4 was used at a concentration of 20 μg/ml, which corresponds to about 400 IU/ml of anti-HPA-1a antibody activity as measured by quantitative mAb immobilization of platelet antigens (MAIPA) assay [24]. mAb 26.4 significantly inhibited adhesion and migration of HTR8/SVneo cells to vitronectin-coated membranes by 15–20% (Fig. 2a and b) and 18–23% (Fig. 2c and d), respectively. Anti-αVβ3 murine mAb (clone LM609) similarly inhibited adhesion and migration of HTR8/SVneo cells (data not shown).

**Fig. 3:**
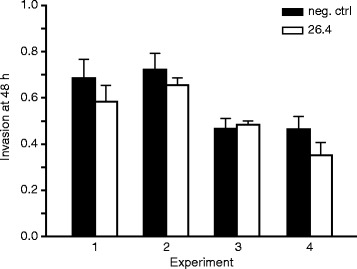
Effect of anti-HPA-1a antibodies on invasive capacity of EVT. HTR8/SVneo cells were cultured in Matrigel coated chambers in the presence of mAb 26.4 or human IgG1 as a negative control for 48 h. The samples were run in quadruplicates and experiment repeated four times. Each column represent the range in cell index of one sample run in quadruplicate. Cell invasion was evaluated by MTT-assay. The invasion data is expressed as the ratio of invasion through the Matrigel Matrix and membrane relative to the migration through the control membrane

The effect of mAb 26.4 on invasive capacity of first trimester trophoblast cells was studied utilizing Matrigel pre-coated invasion chambers. The mAb inhibited invasive capacity of cells in three out of four independent experiments by 9, 15 and 25% (Fig. 3). The inhibition was not statistically significant (*p* = 0.13). Anti-αVβ3 murine mAb (clone LM609) did not affect invasive capacity of HTR8/SVneo cells (data not shown).

### HTR8/SVneo cells originate from a female conceptus

To identify whether HTR8/SVneo cells originate from a female or male conceptus, the Y-chromosome DNA test has been performed. HTR8/SVneo cells were tested negative for Y-chromosome DNA, indicating that the cells originate from a female conceptus.

## Discussion

In the present study, we have demonstrated that a human HPA-1a-specific mAb inhibit adhesion and migratory capacity of EVT cells in an in vitro model.

To study the possible effect of anti-HPA-1a antibodies on EVT we utilized transformed first trimester EVT-derived cells (HTR8/SVneo cell line), which were reported to share phenotypic and functional characteristics of EVT cells [17, 25]. We have shown that HTR8/SVneo cells express HPA-1a epitope as part of αVβ3 integrin complex, and importantly, that anti-HPA-1a antibodies interact with HPA-1a on these cells. In this study, we used a human anti-HPA-1a mAb, clone26.4, generated from a B cell derived from a woman HPA-1a immunized in connection with pregnancy, who had two children affected by FNAIT. mAb 26.4 was expressed recombinantly and found to be highly specific for HPA-1a, bound strongly to HPA-1a epitopes on αIIbβ3 from platelets as well as αVβ3 from trophoblasts [16]. Thus, the HTR8/SVneo cell line with mAb 26.4 could be a useful model to study possible effect of anti-HPA-1a antibodies on EVT.

The idea that alloantibodies reactive with fetal integrins expressed on trophoblast cells can impair placental function is not new. A histological study of placentas from FNAIT-affected pregnancies described chronic villitis in pregnancies not treated with IVIG [26]. And in a recent histopatological study, FNAIT was associated with chronic chorioamnionitis, basal chronic villitis and intervillositis [6]. In addition, a case of FNAIT associated with massive chronic intervillositis has also been described [27]. Chronic villitis and intervillositis are placental lesions known to be associated with poor fetal growth [28, 29]. Further, an association between FNAIT due to anti-HPA-1a antibodies and increased risk of miscarriage has also been suggested, indicating that placental development may be affected in early stages of pregnancy [30, 31].

Vitronectin receptor, carrying HPA-1 antigen epitope, is crucial for cell-matrix and cell-cell interactions, modulating growth, survival, motility and differentiation of angiogenic endothelial cells (EC), osteoclasts, tumor cells and other cell types [32]. Blocking αVβ3 was shown to disrupt the invasive and proliferative program of sprouting EC, and suppress angiogenesis [33–35] impede tumor progression [36], and hinder osteoclast adhesion and migration [32]. The important role of αVβ3 in mediating EVT cells invasion [8] and adhesion to ECs [37, 38] was shown in vitro.

Further, the capacity of anti-HPA-1a antibodies to affect αVβ3-expressing EC in vitro has been reported [35, 39, 40]. Anti-HPA-1a maternal sera affected spreading and monolayer integrity of human umbilical cord endothelial cells (HUVEC) [39] and inhibited HUVEC proliferation and formation of capillary-like networks [35]. The latter findings suggest that anti-HPA-1a antibodies can cause systemic vascular damage, impair angiogenesis, and subsequently can be an independent cause of FNAIT-associated ICH. Further, in a recent study, Santoso S. with co-workers have shown that only anti-HPA-1a antibodies binding selectively to the αVβ3 complex interfere with angiogenesis [40].

Mechanisms of inhibitory effects of anti-HPA-1a antibodies are still incompletely understood. It has been shown that anti-HPA-1a antibodies can impair angiogenic and increase proapoptotic signaling in HUVECs [35]. It has also been hypothesized that anti-HPA-1a IgG antibodies block the ligand RGD binding site on αVβ3 and αIIbβ3 by indirect competition (i.e., steric hindrance) [41].

The HPA-1a epitope is formed by only one amino acid change, L33P, in integrin β3, and, therefore, all anti-HPA-1a antibody epitopes overlap reacting with the L33 residue. Yet, anti-HPA-1a antibodies are reported to be heterogeneous in their footprint on integrin β3 [42] and binding affinity [43–45]. In fact, recently it was found that antibodies of this specificity can be even more complex; αVβ3-, αIIbβ3-specific, or bind antigen independently of the complex [40]. Thus, the effect of a single mAb specific for HPA-1a, as used in this study, may not be representative for different polyclonal antibody profiles among immunized women. Still, our finding that an anti-HPA-1a mAb could affect functions of HTR8/SVneo cells is interesting, indicating that anti-HPA-1a antibodies may affect functions of extravillous trophoblast cells in vivo.

Only male neonates had significantly reduced birth weight in pregnancies with high levels of maternal anti-HPA-1a antibodies in a retrospective observational study [4]. Male sex of the fetus is a well known risk factor for adverse pregnancy outcome [46]. Evidence is emerging on the influence of fetal sex on placental development and function [47]. The placenta displays sexually dismorphic differences in gene expression and responds to maternal factors in a sex-dependent manner [48]. The magnitude of the effects of anti-HPA-1a antibodies on trophoblast cells may depend on the sex of the placenta. In this study we used a cell line HTR8/SVneo which we found to originate from a female placenta. In the follow up studies, it is therefore important to compare the effects of anti-HPA-1a antibodies on trophoblast cells originating from male and female placentas.

## Conclusions

We have demonstrated that a human anti-HPA-1a mAb impaired adhesion and migratory capacity of EVT-derived cell line in vitro. We speculate that anti-HPA-1a antibodies may hinder placental development, and consequently, may be involved in early pregnancy loss as well as poor placental function. Further studies with primary trophoblast cells and maternal anti-HPA-1a sera, together with a histopathological study of placentas from pregnancies affected by FNAIT are important to support our finding.

## Abbreviations

CTB: Cytotrophoblast
EC: Endothelial cells
EVT: Extravillous trophoblast
FNAIT: Fetal and neonatal alloimmune thrombocytopenia
HPA-1a: Human platelet antigen-1a
ICH: Intracranial hemorrhage
WG: Weeks gestation
